# Antibacterial Activity of Alkaloid Fractions from *Berberis microphylla* G. Forst and Study of Synergism with Ampicillin and Cephalothin

**DOI:** 10.3390/molecules21010076

**Published:** 2016-01-11

**Authors:** Loreto Manosalva, Ana Mutis, Alejandro Urzúa, Victor Fajardo, Andrés Quiroz

**Affiliations:** 1 Laboratorio de Productos Naturales, Instituto de la Patagonia, Universidad de Magallanes, Punta Arenas 6210427, Chile; Loreto.manosalva@umag.cl (L.M.); victor.fajardo@umag.cl (V.F.); 2 Laboratorio de Química Ecológica, Departamento de Ciencias Químicas y Recursos Naturales, Universidad de La Frontera, Temuco 4811230, Chile; ana.mutis@ufrontera.cl; 3 Facultad de Química y Biología, Universidad de Santiago de Chile, Casilla 40, Correo 33, Santiago 9170022, Chile

**Keywords:** *Berberis microphylla*, alkaloid extracts, antibacterial activity, synergism

## Abstract

*Berberis microphylla* is a native plant that grows in Patagonia and is commonly used by aboriginal ethnic groups in traditional medicine as an antiseptic for different diseases. The present study evaluated the antibacterial and synergistic activity of alkaloid extracts of *B. microphylla* leaves, stems and roots used either individually or in combination with antibiotics against Gram-positive and Gram-negative bacteria. The *in vitro* antibacterial activities of leaf, stem and root alkaloid extracts had significant activity only against Gram-positive bacteria. Disc diffusion tests demonstrated that the root extract showed similar activity against *B. cereus* and *S. epidermidis* compared to commercial antibiotics, namely ampicillin and cephalothin, and pure berberine, the principal component of the alkaloid extracts, was found to be active only against *S. aureus* and *S. epidermidis* with similar activity to that of the root extract. The minimum inhibitory concentrations (MICs) of the alkaloid extracts ranged from 333 to 83 μg/mL, whereas minimum bactericidal concentrations (MBCs) varied from 717 to 167 μg/mL. In addition, synergistic or indifferent effects between the alkaloid extracts and antibiotics against bacterial strains were confirmed.

## 1. Introduction

Among Berberidaceae, *Berberis* is represented in Chile and Argentina by 20 species [1]. One representative species of the Patagonia region of both countries is *Berberis microphylla* G. Forst, also known as *Berberis buxifolia* and *Berberis heterophylla*, which grows wild in the under-forest, steppe, and forest–steppe ecotones [2]. This species is a perennial shrub with spiny, yellow flowers and black-bluish fruits [3]. The leaves, stems, roots and fruits of this species have been used since ancient times in traditional medicine for treating fever, inflammation, stomachache, diarrhea, urinary tract infection, throat infection, gingivitis, and liver problems [4]. Moreover, the fruit of the plant has been used by the Kawésqar people as food, whereas the Aonikenk used the yellow scraping of the bark as tobacco for its hallucinogenic effect, which is probably caused by the presence of berberine [5].

**Figure 1:**
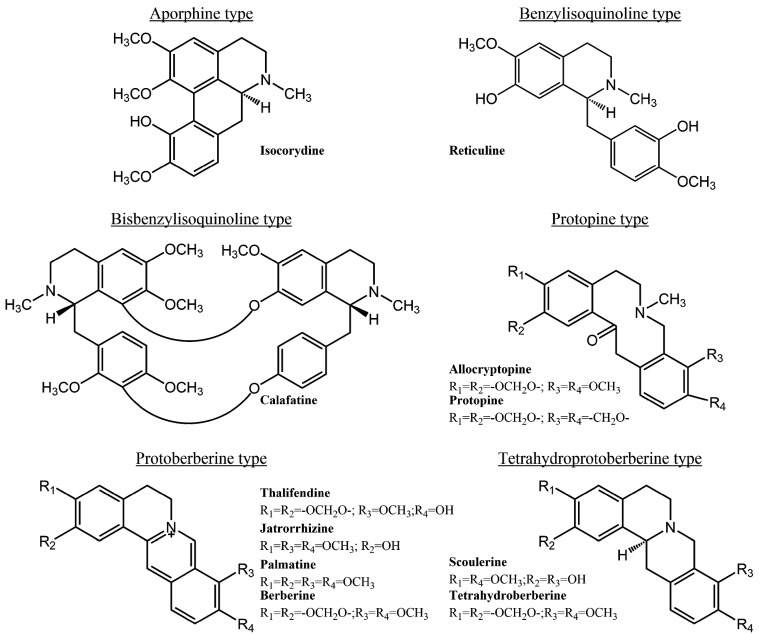
Isoquinoline alkaloids present in leaves, stems and roots of *Berberis microphylla*. Adapted from reference [6].

In previous studies, we have reported that *Berberis microphylla* is a rich source of several types of isoquinoline alkaloids. Leaves, stems and roots contain different alkaloids and different proportions of the shared alkaloids. The root has the highest alkaloid yield, and it contains a complex mixture of the following alkaloids: berberine, allocryptopine, calafatine, jatrorrhizine, palmatine, protopine, reticuline and thalifendine. Stems also contain a complex mixture of the following alkaloids: berberine, allocryptopine, isocorydine, jatrorrhizine, protopine, scoulerine and thalifendine. However, the leaf alkaloid extract contains only berberine and tetrahydroberberine [6] (Figure 1).

Because *B. microphylla* extracts are used in traditional medicine as antiseptics for different diseases, our focus in the present study was to evaluate the antibacterial activity of alkaloid extracts of *B. microphylla* leaves, stems and roots against Gram-positive and Gram-negative bacteria. Furthermore, the fractional inhibitory concentration (FIC) index was used to determine synergy, antagonism, or indifference against the test organisms as a result of interactions between the alkaloid extracts and antibiotics [7].

## 2. Results and Discussion

### 2.1. Antibacterial Activity of Alkaloid Extracts of B. microphylla

*Berberis microphylla* leaf, stem and root alkaloid extracts showed significant antibacterial activity against Gram-positive bacteria but not against Gram-negative bacteria. Susceptibility of Gram-positive bacteria was dependent on the alkaloid extracts tested and the bacterial strain. Pure berberine was found to be active only against *S. aureus* and *S. epidermidis* with similar activity to that of the root extracts (Table 1). It is well known that Gram-negative bacteria have a complex barrier system that can regulate and sometimes prevent the passage of biocides through the cytoplasmic membrane into the cytoplasm [8].

**Table 1:** Inhibition zone diameters (mm) of alkaloid extracts of *Berberis microphylla* against pathogenic bacteria ^a^.

Samples	Microorganisms							
	Gram-Negative				Gram-Positive			
	*E. aerogenes* ATCC 13084	*E.coli* ATCC 25922	*L. monocytogenes* ATCC 13932	*S. typhimurium* ATCC 13311	*S. aureus* ATCC 25923	*B. cereus* ATCC 11778	*S. epidermidis* ATCC 12228	*B. subtilis* ATCC 6633
Plant extracts								
Leaves								
500 µg/disc	i	i	i	i	2.9 ± 0.2 ^h^	2.8 ± 0.2 ^f^	6.4 ± 0.5 ^e^	3.8 ± 0.2 ^g^
1000 µg/disc	i	i	i	i	4.7 ± 0.6 ^g^	4.7 ± 0.5 ^e^	7.7 ± 0.6 ^e^	4.9 ± 0.2 ^ef^
2000 µg/disc	i	i	i	i	7.7 ± 0.2 ^e^	6.9 ± 0.2 ^c^	9.7 ± 0.6 ^d^	5.7 ± 0.6 ^e^
Stems								
500 µg/disc	i	i	i	i	5.7 ± 0.2 ^f^	3.9 ± 0.2 ^e^	7.00 ± 0.0 ^e^	3.8 ± 0.4 ^fg^
1000 µg/disc	i	i	i	i	8.9 ± 0.2 ^d^	5.7 ± 0.6 ^d^	10.7 ± 0.6 ^d^	5.3 ± 0.6 ^e^
2000 µg/disc	i	i	i	i	9.7 ± 0.6 ^d^	7.0 ± 0.5 ^c^	13.0 ± 0.5 ^c^	6.9 ± 0.2 ^cd^
Roots								
500 µg/disc	i	i	i	i	5.7 ± 0.6 ^f^	11.0 ± 0.0 ^b^	9.7 ± 0.2 ^d^	3.8 ± 0.4 ^fg^
1000 µg/disc	i	i	i	i	8.9 ± 0.2 ^d^	12.0 ± 0.5 ^b^	13.0 ± 0.6 ^c^	5.8 ± 0.3 ^de^
2000 µg/disc	i	i	i	i	11.1 ± 0.1 ^c^	13.9 ± 0.2 ^a^	15.3 ± 0.6 ^b^	7.9 ± 0.20 ^c^
Berberine								
500 µg/disc	i	i	i	i	6.9± 0.2 ^e^	i	12.7± 0.2 ^c^	i
1000 µg/disc	i	i	i	i	7.7± 0.2 ^e^	i	14.3± 0.6 ^b^	i
2000 µg/disc	i	i	i	i	9.7± 0.6 ^d^	i	14.9± 0.2 ^b^	i
Antibiotics								
Ampicillin 10 µg/disc	10.0 ± 0.0	21.0 ± 0.0	30.0 ± 0.0	19.0 ± 0.0	34.0 ± 0.5 ^b^	11.0 ± 0.06 ^b^	16.7 ± 0.5 ^b^	27.0 ± 0.06 ^b^
Cephalothin 30 µg/disc	20.0 ± 0.0	16.0 ± 0.0	20.0 ± 0.0	18.0 ± 0.0	35.6 ± 0.06 ^a^	2.6 ± 0.0 ^f^	25.9 ± 0.2 ^a^	36.0 ± 0.06 ^a^

Discount the diameter of a sterile disc (5 mm); i: inactive. ^a^ mean of triplicates ± standard deviation of three replicates; ^b−g^ different letters indicated significant differences according to Tukey test (*p* < 0.05).

Although the preparation of extracts, bacterial strains, fraction concentrations and microbiological techniques used in the literature for determining antimicrobial activity are not standardized, our results are in agreement with these reports. Previous reports have shown that extracts of *Berberis* spp. have antibacterial activities that may be principally associated with the presence of alkaloids in leaves, stems and roots. The root and stem hydroalcoholic extracts of four *Berberis* spp. (*B. aristata*, *B. asiatica*, *B. chitria* and *B. lycium*) are effective against 11 Gram-positive bacterial strains, with berberine as the probable component responsible for antimicrobial activity [9]. Another study showed that the methanolic extract of *B. lyceum* is active against nine of 11 Gram-positive and four of seven Gram-negative bacterial strains [10]. Different extracts of the stem bark of *B. asiatica* were previously tested against 19 bacterial strains; these extracts were active against *Staphylococcus aureus* and *Enterococcus faecalis* but had very little activity against the 11 Gram-negative bacterial strains. In this same study, pure berberine was found to be less active than the tested extracts [11].

The minimum inhibitory concentration (MIC) and minimum bactericidal concentration (MBC) of *B. microphylla* alkaloid extracts are shown in Table 2. The differences in the MIC and MBC values suggest that the *B. microphylla* alkaloid extracts had promising antimicrobial activity. The susceptibility of test organisms was dependent on the alkaloid extracts of the different plant tissues and the bacterial strain. The stem and root alkaloid extracts showed lower MIC and MBC values for *S. epidermidis*. These results may be related to the complexity of the mixture of alkaloids (synergistic effect) in the stem and root as well as the presence of the inactive antimicrobial tetrahydroberberine in the alkaloid extract of leaves [6,7,8,12]. This is the first report on the antibacterial properties of the alkaloid extracts of *B. microphylla*.

**Table 2:** Minimum inhibitory concentrations (MICs) ^a^ and minimum bactericidal concentrations (MBCs) ^a^ of alkaloid extract of *Berberis microphylla* and berberine against Gram-positive bacteria.

Samples	Gram-Positive Bacteria							
	*S. aureus* ATCC 25923		*B. cereus* ATCC 11778		*S. epidermidis* ATCC 12228		*B. subtilis* ATCC 6633	
	MIC	MBC	MIC	MBC	MIC	MBC	MIC	MBC
**Plant Extract**								
Leaves	250 ± 0	750 ± 0	333 ± 118	717 ± 118	125 ± 0	250 ± 0	333 ± 118	717 ± 118
Stems	167 ± 50	334 ± 100	125 ± 0	250 ± 0	83 ± 30	167 ± 60	250 ± 0	500 ± 0
Roots	83 ± 30	167 ± 60	125 ± 0	250 ± 0	83 ± 30	167 ± 60	167 ± 50	334 ± 100
**Berberine**	167 ± 50	334 ± 100	i	i	167 ± 50	334 ± 100	i	i

^a^ MIC and MBC values given as µg/mL, mean of triplicates ± standard deviation of three replicates. i: inactive.

Other antibacterial studies of plant alkaloid extracts have shown similar MIC and MBC values with variances according to bacteria and plant compounds. Extracts are classified as antimicrobials on the basis of MICs in the range of 100–1000 µg/mL [13]. Alkaloid extracts of the aerial part of *Sida acuta* had MIC and MBC values against different clinical isolates of *Staphylococcus* that varied from 80 to >400 μg/mL [14]. Berberine, jatrorrhizine and the crude extract of *Mahonia aquifolium* showed activity against 20 clinical isolates of *Propionibacterium acnes*. The alkaloid extracts of *Mahonia aquifolium* showed MIC values that varied from 100 to 500 μg/mL against *Staphylococcus epidermidis* and from 250 to 500 μg/mL against *S. hominis* [15].

In addition to the known antibacterial activity of berberine, jatrorrhizine and tetrahydroberberine [6,7,8,12], the antibacterial activity of other pure alkaloids found in *B. microphylla* alkaloid fractions have been previously reported. Protopine and allocryptopine, isolated from aerial parts of *Hypecoum erectum* L., showed significant antibacterial activity (MIC 125 μg/mL) against the Gram-negative bacteria *Escherichia coli* and *Pseudomonas aeruginosa* and low antibacterial activity (MIC > 500 μg/mL) against the Gram-positive bacteria *Staphylococcus aureus*, *Bacillus cereus* and *B. subtilis* [16]. Tetrandrine and demethyltetrandrine, two bisbenzylisoquinoline alkaloids isolated from *Stephania tetrandra* S. Moore, related to calafatine, showed *in vitro* anti-MRSA and antibiotic synergistic effects with four antibiotics: ampicillin, azithromycin, cefazolin and levofloxacin [17]. Also, reticuline isolated from *Annona salzmanni* D.C. tested against the Gram-positive and Gram-negative bacteria: *Staphylococcus aureus*, *Bacillus cereus*, *B. subtilis*, *Escherichia coli* and *Pseudomonas aeruginosa* showed to be inactive [18]. Protopine, allocryptopine, calafatine and reticuline are present in low proportion in root alkaloid extracts, and stem alkaloid extracts contain only protopine and allocryptopine [6]. The presence of these compounds in the root and stem alkaloid extracts of *B. microphylla* is not reflected in significant changes of the MIC values of these fractions (Table 2).

### 2.2. Fractional Inhibitory Concentration Index

Synergistic interactions are a result of a combined effect of active compounds from extracts and antibiotics [19]. Antimicrobial compounds from plants may inhibit bacteria by several alternative mechanisms *vs.* antibiotics, enhancing the activity of the latter.

Synergy research in phytomedicine has established synergy as being important [20]. Several examples of synergistic activity between natural plant compounds and conventional antibacterial agents as an alternative way of overcoming resistance of pathogenic bacteria to current antibiotics have been reported [21,22,23].

Based upon FIC index calculations, the combination of alkaloid extracts of *B. microphylla* and ampicillin (AMP) and cephalothin (CFL) showed synergistic and indifferent effects against pathogenic bacteria, respectively (Table 3 and Table 4). A synergistic effect was observed against *B. cereus*, *B. subtilis* and *S. epidermidis* with the leaf alkaloid extract/AMP combination. This effect was also observed against *S. aureus* and *S. epidermidis* with the stem alkaloid extract/AMP combination, and the root alkaloid extract/AMP combination showed the same effect against *B. subtilis* (Table 3). The synergistic effect caused by alkaloid extracts and CFL was observed with the following combinations: leaf alkaloid extract/CFL against *S. aureus*, *B. cereus* and *S. epidermidis*; stem alkaloid extract/CFL against *S. aureus* and *B. subtilis*; and root alkaloid extract/CFL against *B. cereus* and *S. epidermidis*.

The synergistic effects of *B. microphylla* alkaloid extracts in combination with AMP and CFL against Gram-positive bacterial strains were in agreement with previous studies on isoquinoline alkaloids. The isoquinoline alkaloid-rich extracts of *Stephania suberosa* also show synergistic effects when combined with AMP against AMP-resistant *Staphylococcus aureus* [24]. The synergistic and additive antimicrobial activities of the bisbenzylisoquinoline alkaloids, namely tetrandrine and demethyltetrandrine, show that both compounds enhance the inhibitory efficacy of cefazolin antibiotics against methicillin-resistant *S. aureus in vitro* [17]. Berberine, which is the principal component of *B. microphylla* alkaloid extracts, has been shown to enhance the antibacterial activity of selected antibiotics against coagulase-negative *Staphylococcus* strains *in vitro* [25].

## 3. Experimental Section

### 3.1. Plant Material

Representative samples of leaves, stems and roots of *Berberis microphylla* were collected during the flowering season at Lago Deseado, Province of Tierra del Fuego (54°22′12.4′′ S; 68°45′45.0′′ W) in December of 2011. A voucher specimen was deposited at the herbarium of the Universidad de Concepcion (Voucher No CONC 178057). The plant material was vacuum-packed and stored at −20 °C for further study.

### 3.2. Alkaloid Extraction

Extraction was performed according to a previously described method [26] with some modifications [6]. In brief, oven-dried and powdered leaves (50 g), stems (50 g) and roots (50 g) of *B. microphylla* were sequentially extracted (24, 48 and 72 h) with methanol at room temperature. The pooled methanolic extracts of each plant tissue were evaporated *in* *vacuo* at 40 °C, and the residues were agitated with 100 mL of 10% HCl for 1 h, incubated for 12 h at 10 °C and then filtered. The filtrates were washed with CHCl_3_ (5 × 80 mL). The CHCl_3_ washings yielded brown non-alkaloidal extracts upon evaporation, which were not investigated. The aqueous phases were adjusted to pH 10 with NH_4_OH and extracted with CHCl_3_ (5 × 80 mL). The solvent was evaporated to obtain the alkaloid extracts of the roots, stems and leaves. The alkaloid compositions of the fractions were identical to those previously published [6].

**Table 3:** Fractional inhibitory concentration index (FICI); combination of alkaloid extract of *Berberis microphylla* with ampicillin.

Samples	Gram-Positive																			
	*S. aureus* ATCC 25923					*B. cereus* ATCC 11778					*S. epidermidis* ATCC 12228					*B. subtilis* ATCC 6633				
	MICa	MICb	FIC	FICI	Effect	MICa	MICb	FIC	FICI	Effect	MICa	MICb	FIC	FICI	Effect	MICa	MICb	FIC	FICI	Effect
Leaves extracts (µg/mL)																				
Leaves	250	125	0.5	1	I	358	89.5	0.25	0.5	S	124	31.0	0.25	0.5	S	333	83.25	0.25	0.5	S
Ampicillin	0.06	0.03	0.5			4.0	1.0	0.25			1.6	0.4	0.25			0.03	0.0075	0.25		
Stems extracts (µg/mL)																				
Stems	167	41.7	0.25	0.5	S	125	125	1	2	I	83	41.5	0.5	1	I	274	68.5	0.25	0.5	S
Ampicillin	0.06	0.015	0.25			4.0	4.0	1			1.6	0.8	0.5			0.03	0.0075	0.25		
Roots extracts (µg/mL)																				
Roots	83	41.5	0.5	1	I	125	125	1	2	I	83	41.5	0.5	1	I	187	46.7	0.25	0.5	S
Ampicillin	0.06	0.03	0.5			4.0	4.0	1			1.6	0.8	0.5			0.03	0.0075	0.25		

MICa: MIC of sample alone; MICb: MIC of the combination. FIC of alkaloid extracts: MIC of alkaloid extract in combination with antibiotic/MIC of alkaloid extract. FIC of antibiotic: MIC of antibiotic in combination with alkaloid extracts/MIC of antibiotic. FICI index: FIC of alkaloid extract + FIC of antibiotic. S: Synergistic; I: Indifferent.

**Table 4:** Fractional inhibitory concentration index (FICI); combination of alkaloid extract of *B. microphylla* with cephalothin.

Samples	Gram-Positive																			
	*S. aureus* ATCC 25923					*B. cereus* ATCC 11778					*S. epidermidis* ATCC 12228					*B. subtilis* ATCC 6633				
	MICa	MICb	FIC	FICI	Effect	MICa	MICb	FIC	FICI	Effect	MICa	MICb	FIC	FICI	Effect	MICa	MICb	FIC	FICI	Effect
Leaves extracts (µg/mL)																				
Leaves	250	62.5	0.25	0.5	S	358	89.5	0.25	0.5	S	124	31.0	0.25	0.5	S	333	166.5	0.5	1	I
Cephalothin	0.06	0.015	0.25			50	12.5	0.25			0.2	0.05	0.25			0.01	0.005	0.5		
Stems extracts (µg/mL)																				
Stems	167	41.7	0.25	0.5	S	125	62.5	0.5	1	I	83	41.5	0.5	1	I	274	68.5	0.25	0.5	S
Cephalothin	0.06	0.015	0.25			50	25	0.5			0.2	0.1	0.5			0.01	0.0025	0.25		
Roots extracts (µg/mL)																				
Roots	83	41.5	0.5	1	I	125	31.2	0.25	0.5	S	83	20.7	0.25	0.5	S	187	93.5	0.5	1	I
Cephalothin	0.06	0.03	0.5			50	12.5	0.25			0.2	0.05	0.25			0.01	0.005	0.5		

MICa: MIC of sample alone; MICb: MIC of the combination. FIC of alkaloid extracts: MIC of alkaloid extract in combination with antibiotic/MIC of alkaloid extracts. FIC of antibiotic: MIC of antibiotic in combination with alkaloid extracts/MIC of antibiotic. FICI index: FIC of alkaloid extracts + FIC of antibiotic. S: Synergistic; I: Indifferent.

### 3.3. Microorganism Strains and Antibiotics

The alkaloid extracts of *B. microphylla* were tested against representative Gram-negative and Gram-positive bacteria. The following bacteria were tested: *Escherichia coli* (ATCC 25922), *Salmonella typhimurium* (ATCC 13311), *Listeria monocytogenes* (ATCC 13932), *Enterobacter aerogenes* (ATCC 13084), *Staphylococcus aureus* (ATCC 25923), *Bacillus cereus* (ATCC 11778), *Staphylococcus epidermidis* (ATCC 12228) and *Bacillus subtilis* (ATCC 6633). AMP, CFL and berberine were purchased from Sigma Aldrich (St. Louis, MO, USA), Oxoid Microbiology Products (Basingstoke, Hants, UK) and United States Biological (Swampscott, MA, USA).

### 3.4. Antibacterial Assays

The susceptibility tests were performed by the Mueller–Hinton agar-well diffusion method. The bacterial strains were grown in Muller–Hinton broth at 37 °C for 12 h and adjusted to a turbidity of 0.5 McFarland standard (1 × 10^8^ CFU/mL). To obtain the inoculum, these suspensions were diluted 100-fold in Muller–Hinton broth to give 1 × 10^6^ CFU/mL.

### 3.5. Antimicrobial Activity (Disc Diffusion Assay)

The antibacterial activities of the alkaloid extracts of *B. microphylla* were assayed against Gram-negative and Gram-positive bacteria using the disc diffusion method recommended previously by the National Committee for Clinical Laboratory Standard (CLSI 2001) and reported by Konaté *et al.* [20] with slight modifications. Briefly, a suspension of the tested microorganism (0.1 mL of 1 × 10^6^ CFU/mL) was spread on solid media plates. The alkaloid compounds of *B. microphylla* were dissolved in 10% dimethyl sulfoxide (DMSO) in water, and the alkaloid solution was filtered through 0.22 μm Millipore Express^®^ (Billerica, MA, USA) membranes for sterile filtration. Filter paper discs (5 mm in diameter) were impregnated with 10 μL of the extracts (equivalent to 500, 1000 and 2000 µg/disc) and placed on the inoculated agar plates. The plates were incubated at 37 °C for 24 h. Discs containing AMP (10 μg) and CFL (30 µg) were used as positive controls, and 10% DMSO was used as a negative control.

### 3.6. Minimum Inhibitory Concentration (MIC)

Determinations of the MIC for AMP, CFL and *B. microphylla* alkaloid extracts against bacterial strains were performed using the broth dilution method [27]. Briefly, bacterial suspensions were adjusted to 1 × 10^8^ CFU/mL, with the aim to achieve 1 × 10^6^ CFU/mL. The diluted inoculum (0.1 mL) of each stain was added to 0.9 mL of Mueller–Hinton broth serial dilutions of the antibacterial agents to give a final concentration of approximately 1 × 10^5^ CFU/mL. The antibiotics and *B. microphylla* alkaloid extracts were dissolved in sterile distilled water to obtain stock solutions of 100 μg/mL for antibiotics or 1000 µg/mL for the extracts. All tests were performed in triplicate and incubated at 37 °C for 24 h. The MIC was defined as the lowest concentration at which no visible growth was observed [28].

### 3.7. Minimum Bactericidal Concentration (MBC)

The MBC was defined as the lowest concentrations of AMP, CFL and *B. microphylla* alkaloid extracts that inhibit the growth of the inoculum by 99.9% within 24 h of incubation at 37 °C [29]. Each experiment was repeated at least three times. The MBC values were determined by removing 100 μL of bacterial suspension from the subculture demonstrating no visible growth and inoculating nutrient agar plates. Plates were incubated at 37 °C for a total period of 24 h.

### 3.8. Determination of the Fractional Inhibitory Concentration (FIC) Index

The combined effect of alkaloid extracts of *B. microphylla* with antibiotics against bacterial strains was evaluated by the mean determination of the fractional inhibitory concentration index with a series of combinations corresponding to 1, 1/2, 1/4, 1/8, 1/16, 1/32, 1/64, and 1/128 of the MIC values after incubation at 37 °C for 24 h. The FIC index for all the combinations was determined using the following formula [17]:

FIC index = FIC_A_ + FIC_B_ = [A]/MIC_A_ + [B]/MIC_B_

where FIC_A_ and FIC_B_ represent the fractional inhibitory concentrations of drugs A and B, respectively; MIC_A_ and MIC_B_ represent the minimum inhibitory concentrations of drugs A and B, respectively; and [A] and [B] represent the concentrations of drugs A and B, respectively.

The FIC index is based on the Loewe additivity zero interaction theory. This theory is based on the hypothesis that a drug cannot interact with itself and therefore the effect of a self-drug combination will always be additive, with an FIC index of 1. An FIC index lower or higher than 1 indicates, respectively, synergy or antagonism. The FIC index as evaluated by the checkerboard method is interpreted as follows: ≤0.5 represents synergy; >0.5 and ≤4 represents additivity/indifference; and >4 represents antagonism [7].

### 3.9. Statistical Analysis

All tests were performed in triplicate, and the bacterial activity was expressed as the mean of inhibition diameters (mm) produced (excluding disc diameter of 5 mm). Inhibition data were checked for normal distribution and variance homogeneity, and the data were analyzed by ANOVA followed by HSD-Tukey’s test for mean separation (*p* < 0.05) using R-commander 2.0.3.

## 4. Conclusions

Our results reveal that the alkaloid extracts of *B. microphylla* leaves, stems and roots present selective antibacterial activity against Gram-positive bacterial strains. Pure berberine, the principal component of the alkaloid extracts [6], was found to be active only against *S. aureus* and *S. epidermidis* with similar activity to that of the root extracts (Table 1). These findings correlate with the use of the plant by aboriginal ethnic groups in traditional medicine as an antiseptic for different diseases. Additionally, based on FIC index calculations, *B. microphylla* alkaloid extracts showed synergistic effects with ampicillin (AMP) and cephalothin (CFL) only on some of the tested bacterial strains. Considering that the synergism observed between alkaloid extracts of *B. microphylla* and antibiotics would reduce the side effects caused by each of these antibacterial agents alone, further research can be focused in the evaluation of toxicity of these alkaloids for eventual future clinical applications.
